# Prevalence of Spina Bifida among Newborns in Africa: A Systematic Review and Meta-Analysis

**DOI:** 10.1155/2020/4273510

**Published:** 2020-10-06

**Authors:** Mohammed Oumer, Molla Taye, Hailu Aragie, Ashenafi Tazebew

**Affiliations:** ^**1**^ Department of Human Anatomy, School of Medicine, College of Medicine and Health Sciences, University of Gondar, Gondar, Amhara, Ethiopia; ^**2**^ Department of Epidemiology, Institute of Public Health, College of Medicine and Health Sciences, University of Gondar, Gondar, Amhara, Ethiopia; ^**3**^ Departments of Pediatrics and Child Health, School of Medicine, College of Medicine and Health Sciences, University of Gondar, Gondar, Amhara, Ethiopia

## Abstract

Spina bifida is an abnormal closure of the neural tube during the fourth week of development. It is the major cause of fetal loss and considerable disabilities in newborns. The aim of this review is to determine the pooled prevalence of spina bifida among newborns in Africa. PubMed/Medline, Google Scholar, Science Direct, Joanna Briggs Institute (JBI) Library, Cochrane Library, Web of Science, African Journals Online, and Embase databases were systematically searched. Cochran Q test and *I*^2^ test statistics were applied to assess heterogeneity across studies. A random-effect model was applied to calculate the pooled prevalence of spina bifida. Forest plot and Galbraith's plot were used to visualize heterogeneity. Subgroup, sensitivity, meta-regression, and meta-cumulative analyses were performed. All essential data were extracted using a standardized data extraction format, and the JBI quality appraisal checklist was used to assess the quality of studies. Egger's test and Begg's test were used in order to detect the publication bias. In the present systematic review and meta-analysis, 6,587,298 births in twenty-seven studies were included. The pooled birth prevalence of spina bifida in Africa was 0.13% with a range between 0.12% and 0.14%. In Africa, the highest burden of spina bifida was detected in Algeria (0.43%), Ethiopia (0.32%), Tanzania (0.26%), Cameron (0.12%), Egypt (0.10%), and South Africa (0.10%). The lowest burden of spina bifida was detected in Libya (0.006%) and Tunisia (0.009%). The high birth prevalence of spina bifida was detected in Africa. There was a significant variation in the prevalence of spina bifida among study countries in Africa. The authors recommend that special awareness creation with the help of health education intervention should be provided for mothers to focus on prevention in order to reduce the burden of spina bifida.

## 1. Introduction

Spina bifida is an abnormal closure of the neural tube during the fourth week of development [1–4]. Spina bifida is a general term for neural tube defects affecting the spinal cord [2, 5–9]. It consists of splitting of the vertebral arches and may or may not involve underlying neural tissue [2, 10]. Spina bifida ranges from clinically significant types to minor anomalies that are functionally unimportant [1]. Spina bifida occulta and cystica are the main types of spina bifida [1, 2]. Spina bifida occulta is a failure of fusion of the vertebral arches that are covered by the skin and mostly does not involve the spinal cord [10–14]. Most often, the defect occurs in the sacral area, from S1 to S2, and is usually marked by a patch of hair overlying the affected region [2, 14]. Often, the defect is detected when an X-ray of the back is performed before birth [2]. Spina bifida cystica is a severe type of spina bifida in which a neural tissue protrudes through an opening in the vertebral arches to form a cyst-like sac. It happens at any site of the vertebral column, but the most common site of the protrusion is the lumbosacral area [1, 2, 14–18]. Sometimes, only covering membrane or meninges (with cerebrospinal fluid) herniate through the defect, which is referred to as spina bifida with meningocele; in another way, neural tissue is included in the sac, and the anomaly is known as spina bifida with meningomyelocele. Meningomyelocele is a more common and severe type of spinal bifida cystica that is often associated with a marked neurologic deficit inferior to the level of the protruding sac [1, 3]. Myeloschisis is the most severe type of spina bifida. Myeloschisis occurs when the neural folds remain as a flattened mass of neural tissue [2]. Spina bifida with myeloschisis may result from a defect that is caused by a local overgrowth of the neural plate. As a result, the caudal neuropore fails to close at the end of the fourth week [1, 6]. Due to the tethering of the spinal cord, the incidence of hydrocephaly is expected to be very high in each case of spina bifida cystica [2, 3]. It shows different levels of neurologic deficit based on the position and extent of the lesion [1, 4].

It is the major cause of fetal loss and considerable disabilities in newborns, and it is considered a significant public health problem [3, 4]. Spina bifida occurs in approximately one in every thousand births. However, the birth prevalence of spina bifida varies among different populations and as high as one in every hundred births in some areas, such as northern China. The incidence of spina bifida shows a geographic variation in incidence. In the British Isles, the occurrence of the defect varies from 1.5 per thousand births in southeastern England to 4.2 per thousand births in South Wales [1, 2]. In the United States of America, the estimate of spina bifida has reduced, by twenty-five percent, to one per 1,500 births since the adaptation of the fortification of flour with folic acid [2].

Spina bifida can be diagnosed prenatally by imaging techniques such as ultrasound and by identification of alpha-fetoprotein levels in the amniotic fluid or serum. Any malformation or abnormality in the vertebral arches is starting to be visualized by twelve weeks of gestation [2, 7].

The origin of most spina bifida malformations is multifactorial, and the probability of having a baby with such a malformation increases significantly once an affected baby is born. Recent evidence proves that folic acid decreases the incidence of spina bifida by as much as fifty to seventy percent if four hundred micrograms are taken daily beginning a month prior to the conception and continuing through an early gestation. Since about fifty percent of all pregnancies are unplanned, it is recommended that all women of childbearing age take multivitamins containing four hundred micrograms of folic acid daily. In addition, a mother with a history of a baby with a defect or a family history of the defect should take four hundred micrograms of folic acid daily and then four thousand micrograms per day starting at least one month before the conception through the first three months of pregnancy [2, 14].

The aim of this systematic review and meta-analysis was to determine the pooled birth prevalence of spina bifida among newborns in Africa.

## 2. Materials and Methods

### 2.1. Reporting of the Findings and Systematic Review Registration

The preferred reporting items for systematic reviews and meta-analysis (PRISMA) statements were adapted to report this systematic review and meta-analysis [19]. The protocol has been registered on an International Prospective Register of Systematic Review (PROSPERO), University of York Center for Reviews and Dissemination (https://www.crd.york.ac.uk/) (registration number ID: 167659).

### 2.2. Searching Strategies

The presence of systematic review and meta-analysis on the prevalence of spina bifida in Africa was checked on different databases (DARE database, Cochrane Library, and JBI Library) to avoid duplication. PubMed/Medline, Cochrane Library, JBI Library, CINAHL, POPLINE, Science Direct, Web of Science, African Journals Online, WHO, UCSF, and Embase databases were systematically searched for relevant studies. Reference lists (bibliographies) of identified studies were also checked for the presence of additional studies. Grey literature was retrieved using Google and Google Scholar searches. The primary search was conducted in an advanced PubMed database: (Prevalence) AND (“neural tube defects” OR “neural tube defects” [MeSH Terms] OR “spina bifida”) AND (newborns OR neonate [MeSH Terms]) AND (Africa). These core search terms and phrases were considered interchangeably in different databases. The last search was conducted on March 22, 2020.

### 2.3. Eligibility Criteria

#### 2.3.1. Inclusion Criteria

The inclusion criteria for this review were published and unpublished full articles done in any period, articles reported in the English language, and any type of study designs that report the prevalence of spina bifida at birth within Africa.

#### 2.3.2. Exclusion Criteria

Abstracts, anonymous reports, conferences, editorials, case reports, and articles without full access (after contacting the corresponding author two times through e-mail) were excluded from the review. In addition, a study was excluded if the total number of cases as well as the total number of births included in the study were not explicitly stated.

### 2.4. Study Outcome

The outcome of this review was the pooled birth prevalence of spina bifida. Birth prevalence of spina bifida is defined as the number of spina bifida cases of live births or stillbirths at birth from the total number of births during the study period.

### 2.5. Validity Assessment

The quality of each study was assessed by the JBI quality appraisal checklist [20]. This critical appraisal checklist was adapted for the studies reporting the prevalence data (it contains nine items from the item appropriateness of the sample frame to the adequacy of the response rate). Two authors independently evaluated the quality of each original study using the tool. Disagreements between authors that arise during criticizing the quality were solved based on evidence-based discussions. The study was considered low risk if the study scored fifty percent and above of all quality assessment items of each study design.

### 2.6. Data Extraction and Study Selection

After retrieving all studies from the databases, they were imported into the reference manager, EndNote version 7 software, to remove the duplicate studies. Then, all eligible study reports were screened by the authors based on the title and abstract for possible inclusion. These studies were deeply reviewed for entire full-text articles to determine the final included article. All needed qualitative and quantitative data were extracted independently by two authors from selected studies using a predetermined standardized data extraction format. The data abstraction format included primary author, publication year, country of the study, sample size, study design, duration of the study, study setting, and the prevalence of spina bifida.

### 2.7. Statistical Analysis

The data analyses were conducted using Stata 11 statistical software. The data were extracted in Microsoft Excel and exported into Stata for further analysis. All study prevalence reports in different denominators were transformed into per hundred births to maintain uniformity.

#### 2.7.1. Meta-Analysis

The heterogeneity between the studies was assessed by using the Cochran Q test statistic, *I*^2^ test statistic, and *P* values. The heterogeneity was considered as low, moderate, or high when *I*^2^ test statistic results were 25%, 50%, and 75%, respectively [21]. This review showed that there was statistically significant heterogeneity among studies (*P* < 0.001). Therefore, a random-effect model was used to estimate the pooled prevalence of spina bifida [22, 23]. Due to the presence of heterogeneity, subgroup analysis was conducted based on selected variables to minimize the variability. Then, a sensitivity analysis was done to observe the influence of a single study on the overall estimation of meta-analysis. Meta-regression analysis was also done to identify the source of heterogeneity among the studies. To observe the random variations in the time sequence among the studies, a time trend analysis (and meta-cumulative analysis) was employed. The forest plot and Galbraith plot were used to visualize the presence of heterogeneity among the studies.

### 2.8. Assessment of the Risk of Bias in Studies

Egger's regression test and Begg's test statistics were applied to detect publication bias objectively [24, 25]. Egger's publication bias plot was used to visualize the publication bias graphically. A statistically significant publication bias was considered if *P* value ≤0.05.

## 3. Results

### 3.1. Study Selection

**Figure 1:**
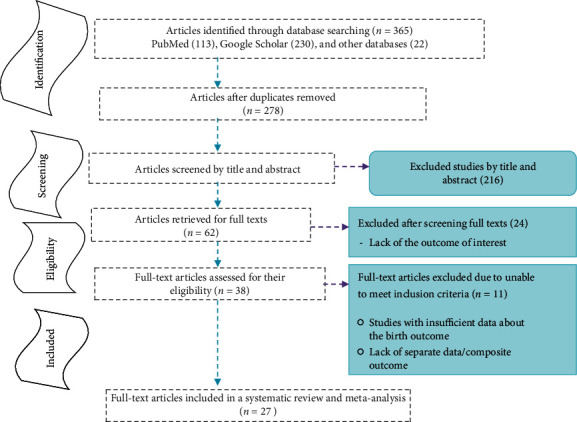
Study selection flow diagram, a figure adapted from the PRISMA group statement.

A total of 365 articles were initially retrieved regarding spina bifida in Africa through PubMed, Google Scholar, and others from Cochrane, JBI Library, WHO, Medline, UCSF, Science Direct, African Journal Online, and Embase. Of these, 87 were excluded due to duplicated articles. From the remaining 278 articles, 216 articles were excluded after reviewing their titles and abstracts because their titles were found irrelevant for this review. The rest 62 articles were screened for the full text, and 24 were excluded due to the outcome of interest (include studies such as risk factor studies, clinical pattern and management studies, and prevention or maternal awareness studies). Therefore, 38 full-text articles were accessed and assessed for eligibility criteria (one study was excluded [26] due to the similarity of the finding from their previous study). Finally, 27 studies fulfilled the eligibility criteria and were included in the systematic review and meta-analysis (Figure 1).

### 3.2. Characteristics of Included Studies

All included studies used cross-sectional and prospective study design [3–13, 27–41]. Of these, twenty studies used cross-sectional and seven studies used prospective study designs. In this systematic review, the 27 studies reported a total of 6,587,298 births. The studies included births, ranging from 956 to 3,803,889 [4, 13]. Of all studies, five were conducted in Ethiopia [3, 8–10, 13], four in Nigeria [5, 11, 30, 31], three in South Africa [35, 38, 39], three in Tunisia [4, 7], two in Ghana [29, 36], and two in Sudan [28, 41]. Besides, study has been conducted from Algeria [6], Eritrea [12], the Democratic Republic (DR) of Congo [27], Libya [32], Egypt [33], Cameron [34], Malawi [37], and Tanzania [40]. All studies were published in the year between 1992 and 2020 (Table 1). Based on the study setting, all studies included in this review were hospital-based studies.

### 3.3. Quality of the Studies

By using JBI quality appraisal criteria, the quality score of the included studies ranged between fifty percent and ninety percent. Therefore, no studies were included that had considerable risk in this review (Table 1).

### 3.4. Meta-Analysis

#### 3.4.1. Prevalence of Spina Bifida

**Figure 2:**
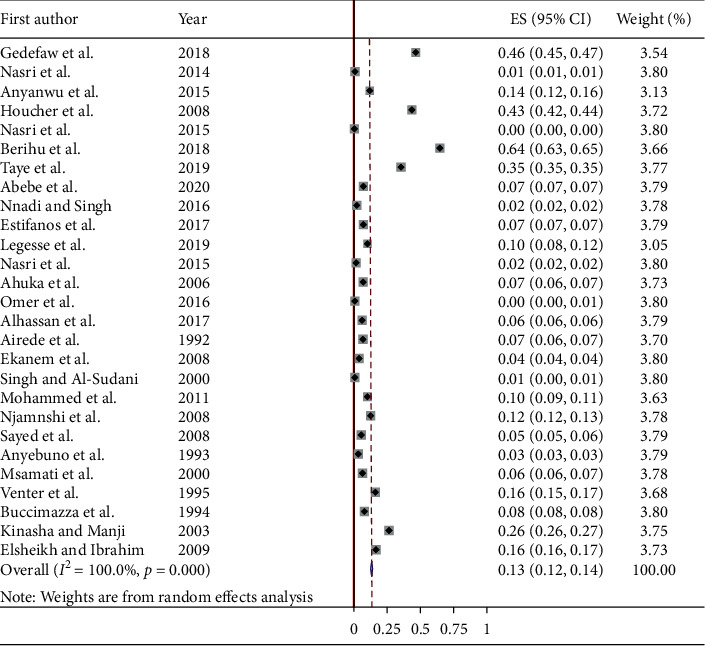
Forest plot showing the pooled prevalence of spina bifida in Africa, 2020.

In this meta-analysis, there was statistically significant heterogeneity across the studies (*P* value <0.001). Therefore, the random-effect model (DerSimonian and Laird's (D + L) method) was applied to estimate the pooled prevalence of spina bifida among studies [3–13, 27–41]. The pooled prevalence of spina bifida was 0.13% (1.3 per 1,000 births). It was ranged in the interval between 0.12% and 0.14% (Figure 2).

**Figure 3:**
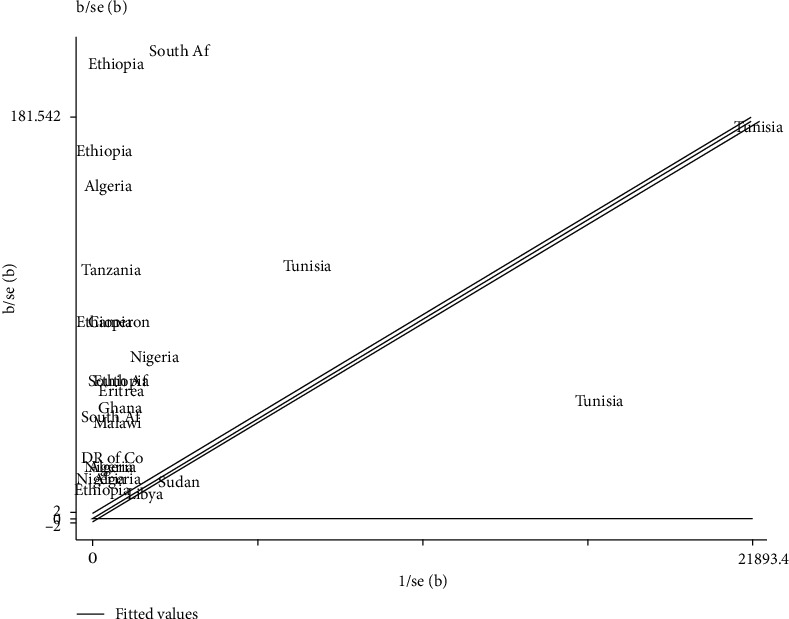
Galbraith plot showing the variability of the pooled prevalence of spina bifida in Africa, 2020.

The Galbraith plot also supported the presence of variation between the studies because most of the study countries were located outside the confidence interval limits (Figure 3).

Subgroup analysis, meta-regression analysis, and sensitivity analysis were the techniques applied to handle variability between the studies.

#### 3.4.2. Subgroup Analysis

Study countries and designs were subgrouped to analyze the pooled prevalence of spina bifida.

*(1) Subgroup Analysis by Country*. In subgroup analysis, statistically significant heterogeneity across the studies in all groups was detected (*P* < 0.001, *I*^2^ = 99-100%). Therefore, DerSimonian and Laird's (D + L) pooled prevalence method was considered because the inverse variance method (I-V) can lead to unreliable estimates. The I-V method assumes that all heterogeneity can be attributed due to the covariates. This assumption may lead to exaggerated type I errors in the presence of residual or unexplained heterogeneity. When countries were considered for subgroup analysis in Africa, the pooled prevalence of spina bifida was 0.43% in Algeria, 0.32% in Ethiopia, 0.26% in Tanzania, 0.12% in Cameron, 0.10% in Egypt, and 0.10% in South Africa. The lowest burden of spina bifida was detected in Libya (0.006%) and Tunisia (0.009%) (Table 2). The difference between the countries was statistically significant (*P* value <0.001).

*(2) Subgroup Analysis by Study Design*. Subgroup analysis based on the study design was performed, and significant variation between study designs was detected. Therefore, based on DerSimonian and Laird's method, the pooled prevalence of spina bifida for cross-sectional was 0.19%, and for prospective cohort design, it was 0.08%. Furthermore, the design was classified, and the pooled prevalence of spina bifida for cross-sectional was 0.20% (95% CI: 0.18%, 0.22%), cross-sectional with the retrospective record review was 0.11% (95% CI: 0.08%, 0.14%), and prospective cohort was 0.08% (95% CI: 0.05%, 0.12%).

#### 3.4.3. Meta-Regression Analysis

In this meta-regression analysis, there was no statistically significant variable for the source of heterogeneity across the studies: sample size (*P* value = 0.44), year of publication (*P* value = 0.89), duration of the study in months (*P* value = 0.65), JBI quality score (*P* value = 0.62), study design (*P* value = 0.85), and study country (*P* value = 0.37).

#### 3.4.4. Sensitivity Analysis

**Figure 4:**
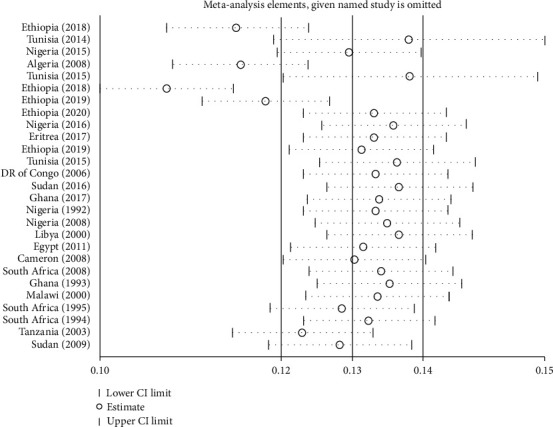
Sensitivity analysis to see the influence of each individual study in Africa, 2020.

There was no individual study especially observed that has a special influence over others. In the full meta-analysis, the influence of studies on pooled estimation was uniform among studies except for four studies (Ethiopia (2018), Algeria (2008), Ethiopia (2018), and Ethiopia (2019)) that showed a minimal influence over others considering that the point estimate of their omitted analysis lies outside the confidence interval of the combined analysis. The graph visually presented the omitted meta-analytic summary statistics as a horizontal confidence interval (the two ends of every broken line), while the full-combined results were displayed as the solid vertical lines (Figure 4). Each circle indicates the pooled prevalence when the left study is omitted in this meta-analysis.

Furthermore, based on this finding, we tried to compute meta-analysis estimates by omitting one study in each turn. Influence analysis is done, turn by turn, by omitting those mentioned studies. Thus, their combined point estimate after omitting the study of Ethiopia (2018), Algeria (2008), Ethiopia (2018), and Ethiopia 2019 was 0.12% (95% CI: 0.11, 0.13%), 0.12% (95% CI: 0.11, 0.13%), 0.11% (95% CI: 0.10, 0.12%), and 0.12% (95% CI: 0.11, 0.13%), respectively. If this removal process is repeated for the other 23 studies, their estimate is similar to the original pooled estimate, 0.13%. However, a reduction in heterogeneity was not observed if the leave-one-out analysis was performed for each study by repeating the whole analysis.

#### 3.4.5. Time Trend Analysis

**Figure 5:**
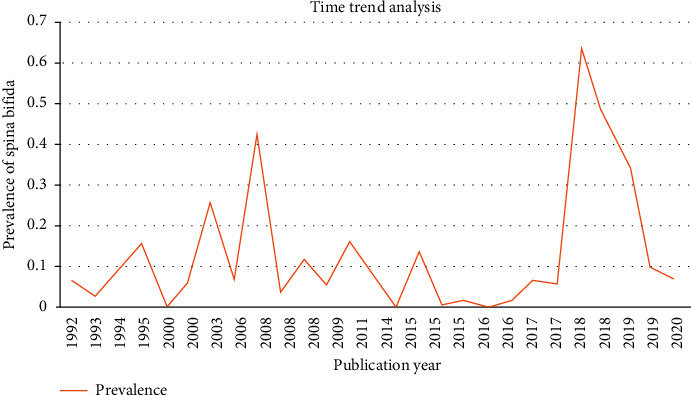
Time trend analysis of the prevalence of spina bifida in relation to the publication year in Africa, 2020.

The time trend analysis showed the relationship between the prevalence of spina bifida with the publication year from 1992 (0.067%) to 2020 (0.070%) (Figure 5). The highest peak of spina bifida was observed between 2017 and 2020.

**Figure 6:**
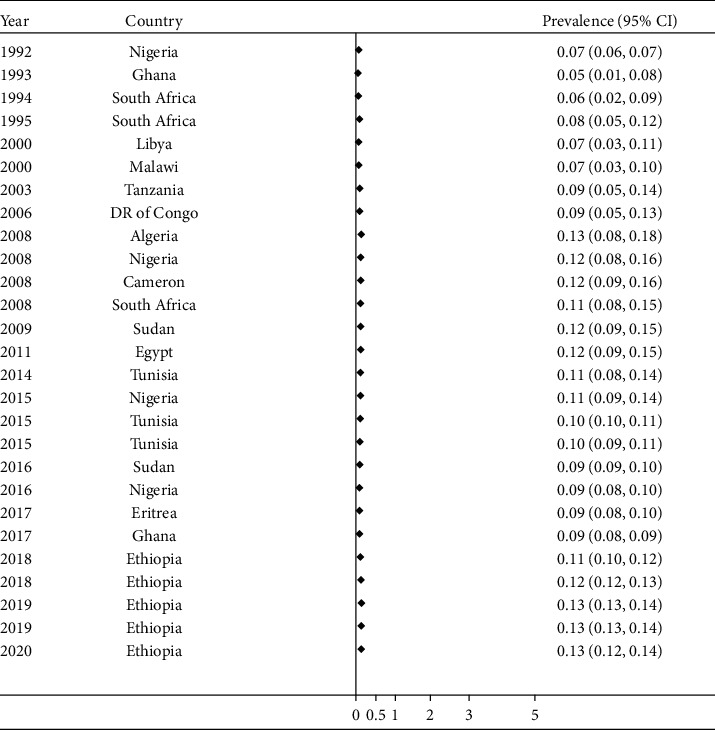
Meta-cumulative analysis of the prevalence of spina bifida in Africa, 2020.

Meta-cumulative analysis showed the cumulative effect of the burden of spina bifida from 1992 (0.07%) to 2020 (0.13%) (Figure 6).

#### 3.4.6. Publication Bias

**Figure 7:**
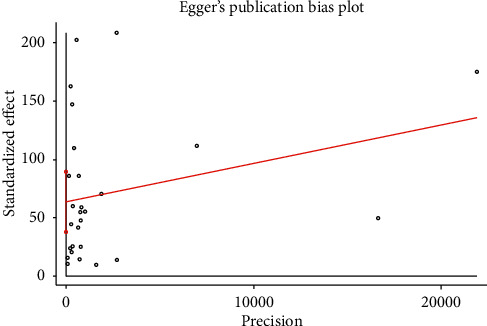
Egger's publication bias plot, 2020.

Statistically significant publication bias in estimating the pooled prevalence of spina bifida was detected by Egger's regression tests (B-coefficient: 63; *P* value<0.001). Egger's regression plot supported its results (Figure 7). However, Begg's test showed as there was no significant publication bias (*P* = 0.381).

**Figure 8:**
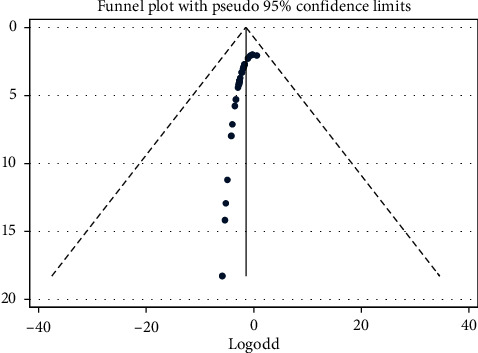
Funnel plot showing the results of the publication bias among studies, 2020.

Essentially, because the effect is proportion, the proportion is transformed into the log-odds scale due to its better statistical properties for meta-analysis as the standard error depends on the value of the log-odds. Nevertheless, the estimates in the funnel plot are less reliable when the proportion is approaching zero (below 5%) given an increment of the standard error, naturally. The result of the funnel plot is presented as shown in Figure 8.

#### 3.4.7. Trim and Filled Meta-Analysis

**Figure 9:**
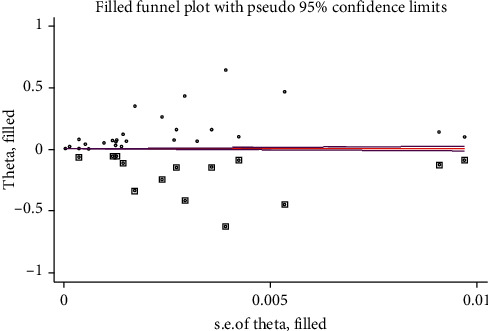
Trim and fill analyses to trim publication bias among studies, 2020.

To handle the publication bias, trim and fill analyses were performed (Figure 9). A fixed-effect model with a linear-type trimming estimator was used to trim the bias. Then, it was filled using a filled meta-analysis. Forty-one studies (fourteen studies were filled) were considered in the filled meta-analysis, and the pooled prevalence of spina bifida in a random-effect model was 0.016% (95% CI: 0.007, 0.024%).

## 4. Discussion

This systematic review and meta-analysis was employed to determine the pooled birth prevalence of spina bifida in Africa. Even if the burden of spina bifida in Africa was high, reviews show that its overall burden was scarce, and attention given to spina bifida seems very low. Therefore, this review provides estimates of the burden of spina bifida in Africa.

In the current meta-analysis, the pooled prevalence of spina bifida was 0.13%, ranging from 0.12% to 0.14%. The review conducted in the Indian, global estimation of spina bifida, and global review [15, 16, 40] supported this finding. The global review that was conducted worldwide estimated that the birth prevalence of spina bifida was 0.113% with a range from 0.008 to 0.17% [40]. This review result suggested that around two hundred thousand newborns were born with neural tube defects each year in low-and middle-income countries [40]. However, this review included only two studies from Africa for different reasons. Representativeness for Africa was decreased due to this situation. According to the Indian systematic review, the pooled prevalence of spina bifida in India was 0.19% of births, ranging from 0.14 to 0.27% [16]. The burden of spina bifida, as reported by the global estimation of spina bifida, was 0.044%, ranging from 0.04 to 0.047% [15]. Other previous evidence also supported our findings that reported spina bifida occurred in at least one per thousand births globally [1–3, 41]. Recent evidence proves that there is a variation in the prevalence of spina bifida from time to time and place to place [17, 42]. Considering this, the prevalence of spina bifida was ranged from one per thousand births to one per five thousand births [2, 14, 17]. Income level and instituted folic acid fortification are the main factors that determine the prevalence of spina bifida among countries [2, 42–45]. Considering heterogeneity, in this review, there is a substantial variation among studies. Even if we used the random-effect model (accounts the amount of variance both within and between the individual studies) to fit this variation, careful consideration of a pooled result during interpretation and focus on the pooled result of subgroup analysis are recommended.

In the present review, a significant difference in the prevalence of spina bifida in different countries of Africa was observed. For instance, a very high (0.43%) pooled prevalence of spina bifida was detected in Algeria. The pooled prevalence of spina bifida in Ethiopia, Tanzania, and Cameron was 0.32%, 0.26%, and 0.12%, respectively. The lowest burden of spina bifida was detected in 0.006% in Libya and 0.009% in Tunisia. The difference in prevalence among countries may be due to the level of the knowledge of mothers about folic acid supplementation or food fortification and difference of countries' health policies regarding folic acid fortification and other preventive measures [3, 17, 42, 43]. For instance, the estimation of spina bifida in the United States of America has reduced by twenty-five percent since the fortification of flour with folic acid was started [2]. A geographic variation in the incidence of spinal bifida was detected in studies [1, 17]. In the British Isles, the occurrence varies from 0.42% in South Wales to 0.15% in southeastern England [1]. Variation in population to population was also observed; in northern China, the prevalence of spina bifida has been observed as high as one in every hundred births [1, 2]. In addition, a review conducted in Latin America reported that there was a wide geographic variation among Latin American countries [17]. Low-income countries should get high attention due to the high burden of spina bifida [42, 46].

Time trend analysis in this review showed that there was variation at a different time in different studies. The trend was not linear, but it was zigzag that shows variation among studies at a different time. The sensitivity analysis suggested that this review finding/estimate is robust because there was no study that has a special influence over others. In meta-regression analysis, sample size, year of publication, duration of the study, JBI quality score, study design, and study country were not the sources of significant heterogeneity. In estimating the pooled prevalence of spina bifida, substantial variation across studies was detected and explained due to differences in study design and study countries. However, it may make statistical power insufficient to detect a statistically significant association which is why these covariates were not the sources of heterogeneity in meta-regression analysis.

Based on the study design, there is a variation between designs in the estimate of pooled prevalence. The change in the magnitude of spina bifida between the cross-sectional and prospective cohort is presumably due to the change in detection methods, sample size, study period/follow-up period, attrition rate, numbers of studies included (twenty studies were cross-sectional), data collection method, or related to documentation of records.

The techniques for testing the publication bias of the between-studies were considered. Nevertheless, the Egger regression test and the funnel asymmetry plot tend to suggest the presence of publication bias. In that case, we performed the trim and fill analyses to minimize the publication bias. When the proportion is approaching zero (below 5%) or a hundred (above 95%), naturally, the standard error will increase. In this case, estimates in the Egger test or funnel plot become biased and less reliable. Furthermore, the Egger et al. regression asymmetry test and the regression asymmetry plot tend to suggest the presence of publication bias more frequently than the Begg approach (an adjusted Kendall rank correlation test). The Egger test detects funnel plot asymmetry by determining whether the intercept deviates significantly from zero in a regression of the standardized effect estimates against their precision. Egger et al. claimed that the test predicts the discordance between meta-analytic results and single large study or trials, but no formal analysis of coverage or power had been performed [47, 48].

All studies in this review were facility-based studies. Therefore, there should be an underestimation of the estimates of spina bifida because the review does not include many home births and stillbirths that are delivered in the community setting.

## 5. Strength and Limitations of the Study

This review provided cumulative evidence on qualitative and quantitative data of spina bifida in Africa. This review should be interpreted based on the following limitations: the review was represented by the studies from the twenty-seven countries due to limited available data about spina bifida. We considered only English-written studies to meticulously evaluate the quality of the studies. The adequacy of the sample size or variability in the sample size may affect the estimated report. Furthermore, the prevalence estimate did not include the terminated pregnancy of spina bifida; this may decrease the prevalence estimates. Moderate publication bias was detected by Egger's regression tests but not Begg's test. For better evidence, trim and fill analyses were performed to confront the publication bias. Therefore, the concerned bodies should use their scientific judgment to use the prevalence of spina bifida reported before or after the trim and fill analyses.

## 6. Conclusion

The present systematic review and meta-analysis showed that the high birth prevalence of spina bifida was detected in Africa. There was a significant variation in the prevalence of spina bifida among countries in Africa. The birth prevalence of spina bifida was very high in Algeria, Ethiopia, Tanzania, and Cameron.

Therefore, the authors recommend that special awareness creation with the help of health education intervention should be provided for mothers to focus on prevention in order to reduce the burden of spina bifida. Limited available data on spina bifida in Africa inform the need for additional primary research that would improve an estimated prevalence of spina bifida and recommend favorable aid policies through maternal education on preventive measures.

## Figures and Tables

**Table 1:** The characteristics of original studies included in the systematic review and meta-analysis, 2020.

First author	Year	Country	Study design	Sample size	Duration (months)	Prevalence (%)	JBI quality status
Gedefaw et al. [3]	2018	Ethiopia	Cross-sectional	8,677	7	0.460	Low risk
Nasri [4]	2014	Tunisia	Cross-sectional^*∗*^	3,803,889	240	0.008	Low risk
Anyanwu et al. [5]	2015	Nigeria	Cross-sectional	1,456	9	0.140	Low risk
Houcher et al. [6]	2008	Algeria	Cross-sectional^*∗*^	28,500	36	0.430	Low risk
Nasri [7]	2015	Tunisia	Cross-sectional	827,113	48	0.003	Low risk
Berihu et al. [8]	2018	Ethiopia	Cross-sectional	14,903	9	0.640	Low risk
Taye et al. [9]	2019	Ethiopia	Cross-sectional	76,201	6	0.350	Low risk
Abebe et al. [10]	2020	Ethiopia	Cross-sectional	45,951	60	0.070	Low risk
Nnadi and Singh [11]	2016	Nigeria	Prospective	10,163	36	0.020	Low risk
Estifanos et al. [12]	2017	Eritrea	Cross-sectional^*∗*^	39,803	24	0.070	Low risk
Legesse et al. [13]	2019	Ethiopia	Prospective	956	7	0.100	Low risk
Nasri [7]	2015	Tunisia	Cross-sectional	764,431	48	0.016	Low risk
Ahuka et al. [27]	2006	DR of Congo	Cross-sectional^*∗*^	8,824	96	0.068	Low risk
Omer et al. [28]	2016	Sudan	Cross-sectional	36,785	12	0.005	Low risk
Alhassan et al. [29]	2017	Ghana	Cross-sectional^*∗*^	35,426	48	0.060	Low risk
Airede [30]	1992	Nigeria	Prospective	5,977	36	0.067	Low risk
Ekanem et al. [31]	2008	Nigeria	Cross-sectional^*∗*^	127,929	276	0.037	Low risk
Singh and Al-Sudani [32]	2000	Libya	Prospective	15,938	12	0.006	Low risk
Mohammed et al. [33]	2011	Egypt	Cross-sectional	5,000	7	0.100	Low risk
Njamnshi et al. [34]	2008	Cameron	Cross-sectional^*∗*^	52,710	120	0.123	Low risk
Sayed et al. [35]	2008	South Africa	Prospective	53,000	9	0.054	Low risk
Anyebuno et al. [36]	1993	Ghana	Cross-sectional^*∗*^	19,094	24	0.031	Low risk
Msamati et al. [37]	2000	Malawi	Cross-sectional^*∗*^	25,562	24	0.063	Low risk
Venter et al. [38]	1995	South Africa	Prospective	10,380	40	0.158	Low risk
Buccimazza et al. [39]	1994	South Africa	Cross-sectional^*∗*^	516,252	240	0.078	Low risk
Kinasha and Manji [40]	2003	Tanzania	Cross-sectional^*∗*^	34,000	24	0.261	Low risk
Elsheikh and Ibrahim [41]	2009	Sudan	Prospective	18,378	12	0.163	Low risk

^*∗*^Cross-sectional study design with the retrospective record review.

**Table 2:** The pooled prevalence of spina bifida among African countries, 2020.

S. no.	Country	Prevalence of spina bifida (%) (95% CI)
1	Algeria	0.43 (0.42, 0.44)
2	Tunisia	0.009 (0.004, 0.014)
3	Nigeria	0.06 (0.04, 0.08)
4	Ethiopia	0.32 (0.12, 0.53)
5	Eritrea	0.07 (0.07, 0.07)
6	DR of Congo	0.07 (0.06, 0.07)
7	Sudan	0.08 (−0.07, 0.24)
8	Ghana	0.05 (0.02, 0.07)
9	Libya	0.006 (0.005, 0.007)
10	Egypt	0.10 (0.09, 0.11)
11	Cameron	0.12 (0.12, 0.13)
12	South Africa	0.10 (0.07, 0.12)
13	Malawi	0.06 (0.06, 0.07)
14	Tanzania	0.26 (0.26, 0.27)
Total	D + L pooled	**0.13 (0.12, 0.14)**

## Data Availability

The datasets used and/or analyzed during the current systematic review and meta-analysis are included the review, as well as available from the corresponding author.
